# Supplementary material for: Differentiation of the bacterial communities associated with Orbicella faveolata across different growth conditions and life-cycle stages
Source: PLoS One. 2025 Nov 7;20(11):e0335445. doi: 10.1371/journal.pone.0335445 (PMC12594401; doi:10.1371/journal.pone.0335445)
Supplement: S1 File — Supplementary Table 1. PERMANOVA for bacterial community structures associated with O. faveolata by (A) settler growth conditions and (B) life-cycle stage. Supplementary Table 2. Functional prediction suggested by FAPROTAX by growth condition. Supplementary Table 3. Kruskal-Wallis by settler growth condition. Supplementary Table 4. Functional prediction suggested by FAPROTAX by life-cycle stage. Supplementary Table 5. Kruskal-Wallis by life-cycle stage. Supplementary Table 6. Read counts obtained for each O. faveolata sample after quality filtering. Supplementary Table 7. Number of ASV presented in each O. faveolata sample. Supplementary Fig. 1. Rarefaction curves at 4800 reads across O. faveolata samples. The curves indicate that sequencing depth was sufficient to capture most of the bacterial diversity within the dataset. (PCF01-PCF15 corresponded to LBOFS; PCF21-PCF32 to adult colonies; PCF34-PCF36 to outplanted settlers and PCF37-PCF39 to wild settlers). Supplementary Fig. 2. Alpha diversity estimated by Shannon index and ANOVA analysis for bacterial communities associated with O. faveolata. A) settler growth conditions and B) life-cycle stages. Supplementary Fig. 3 Functional prediction of bacterial communities associated with O. faveolata lab-bred settlers, according to the FAPROTAX database. Supplementary Fig. 4 Bacterial community composition at class level across all O. faveolata samples. Relative abundances of the 10 major bacterial classes are shown. Rare biosphere is grouped under “Others”. (DOCX) [file pone.0335445.s001.docx]

**SUPPLEMENTARY MATERIAL**

**Supplementary Table 1.** PERMANOVA for bacterial community structures associated with *O. faveolata* by (A) settler growth conditions and (B) life-cycle stage.

**
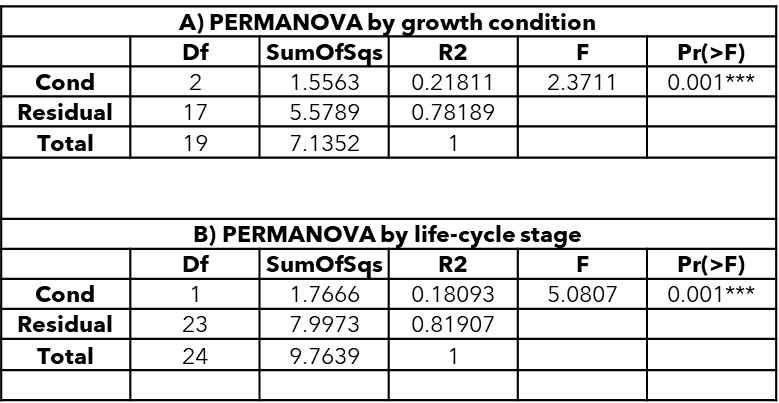
**

**Supplementary Table 2.** Functional prediction suggested by FAPROTAX by growth condition.


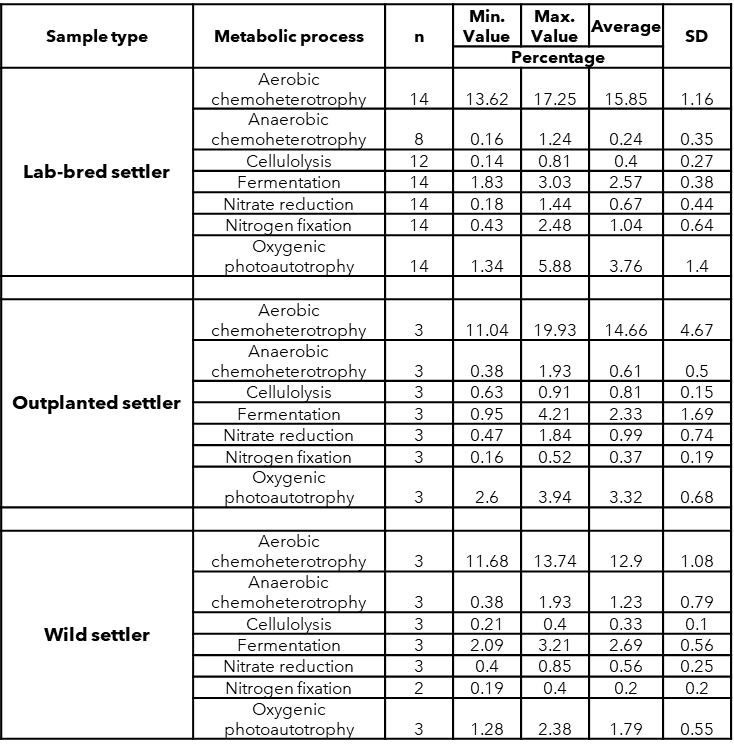


**Supplementary Table 3.** Kruskal-Wallis by settler growth condition.

**Supplementary Table 4.** Functional prediction suggested by FAPROTAX by life-cycle stage.

**Supplementary Table 5.** Kruskal-Wallis by life-cycle stage.

**Supplementary Table 6.** Read counts obtained for each *O. faveolata* sample after quality filtering.

| **ID** | **Read count** | **Sample** |
| --- | --- | --- |
| PCF01 | 13103 | Lab-bred settler |
| PCF02 | 10511 | Lab-bred settler |
| PCF03 | 10438 | Lab-bred settler |
| PCF04 | 8095 | Lab-bred settler |
| PCF05 | 7585 | Lab-bred settler |
| PCF06 | 11516 | Lab-bred settler |
| PCF07 | 12284 | Lab-bred settler |
| PCF08 | 6609 | Lab-bred settler |
| PCF09 | 12500 | Lab-bred settler |
| PCF10 | 11796 | Lab-bred settler |
| PCF11 | 39758 | Lab-bred settler |
| PCF12 | 9468 | Lab-bred settler |
| PCF14 | 6640 | Lab-bred settler |
| PCF15 | 8547 | Lab-bred settler |
| PCF21 | 4800 | Wild adult fragment |
| PCF23 | 8446 | Wild adult fragment |
| PCF26 | 6806 | Wild adult fragment |
| PCF27 | 10234 | Wild adult fragment |
| PCF28 | 11727 | Wild adult fragment |
| PCF29 | 10170 | Wild adult fragment |
| PCF30 | 8069 | Wild adult fragment |
| PCF31 | 6825 | Wild adult fragment |
| PCF32 | 7308 | Wild adult fragment |
| PCF34 | 4936 | Outplanted settler |
| PCF35 | 11139 | Outplanted settler |
| PCF36 | 9872 | Outplanted settler |
| PCF37 | 7135 | Wild settler |
| PCF38 | 16215 | Wild settler |
| PCF39 | 5863 | Wild settler |

**Supplementary Table 7.** Number of ASV presented in each *O. faveolata* sample.

| **ID** | **ASV** | **Sample** |
| --- | --- | --- |
| PCF01 | 475 | Lab-bred settler |
| PCF02 | 594 | Lab-bred settler |
| PCF03 | 729 | Lab-bred settler |
| PCF04 | 442 | Lab-bred settler |
| PCF05 | 446 | Lab-bred settler |
| PCF06 | 763 | Lab-bred settler |
| PCF07 | 676 | Lab-bred settler |
| PCF08 | 556 | Lab-bred settler |
| PCF09 | 729 | Lab-bred settler |
| PCF10 | 537 | Lab-bred settler |
| PCF11 | 896 | Lab-bred settler |
| PCF12 | 380 | Lab-bred settler |
| PCF14 | 474 | Lab-bred settler |
| PCF15 | 535 | Lab-bred settler |
| PCF21 | 431 | Wild adult fragment |
| PCF23 | 295 | Wild adult fragment |
| PCF26 | 488 | Wild adult fragment |
| PCF27 | 311 | Wild adult fragment |
| PCF28 | 919 | Wild adult fragment |
| PCF29 | 712 | Wild adult fragment |
| PCF30 | 577 | Wild adult fragment |
| PCF31 | 491 | Wild adult fragment |
| PCF32 | 351 | Wild adult fragment |
| PCF34 | 1140 | Outplanted settler |
| PCF35 | 726 | Outplanted settler |
| PCF36 | 844 | Outplanted settler |
| PCF37 | 480 | Wild settler |
| PCF38 | 1182 | Wild settler |
| PCF39 | 510 | Wild settler |

**
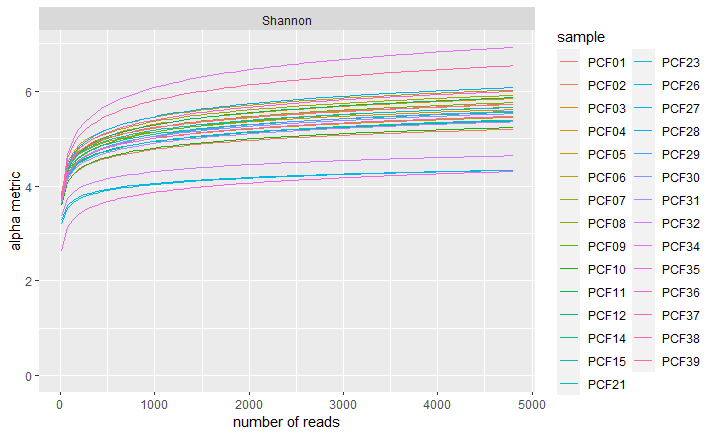
**

**Supplementary Fig. 1.** Rarefaction curves at 4800 reads across *O. faveolata* samples. The curves indicate that sequencing depth was sufficient to capture most of the bacterial diversity within the dataset. (PCF01-PCF15 corresponded to LBOFS; PCF21-PCF32 to adult colonies; PCF34-PCF36 to outplanted settlers and PCF37-PCF39 to wild settlers).


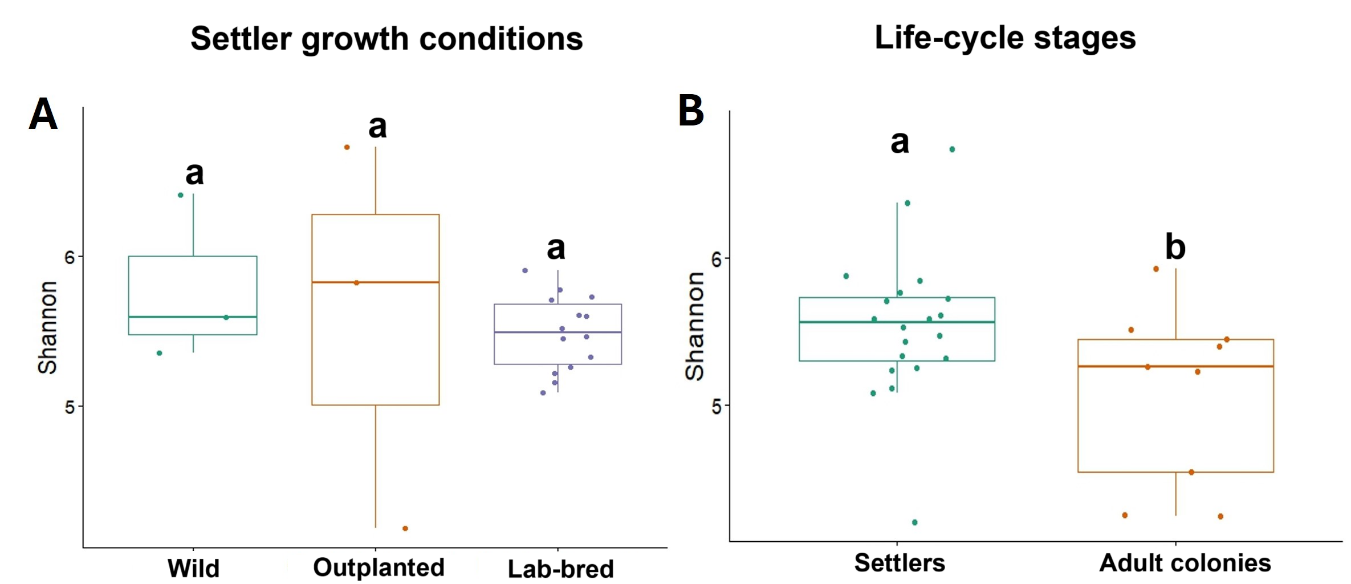


**Supplementary Fig. 2.** Alpha diversity estimated by Shannon index and ANOVA analysis for bacterial communities associated with *O. faveolata.* A) settler growth conditions and B) life-cycle stages.

**
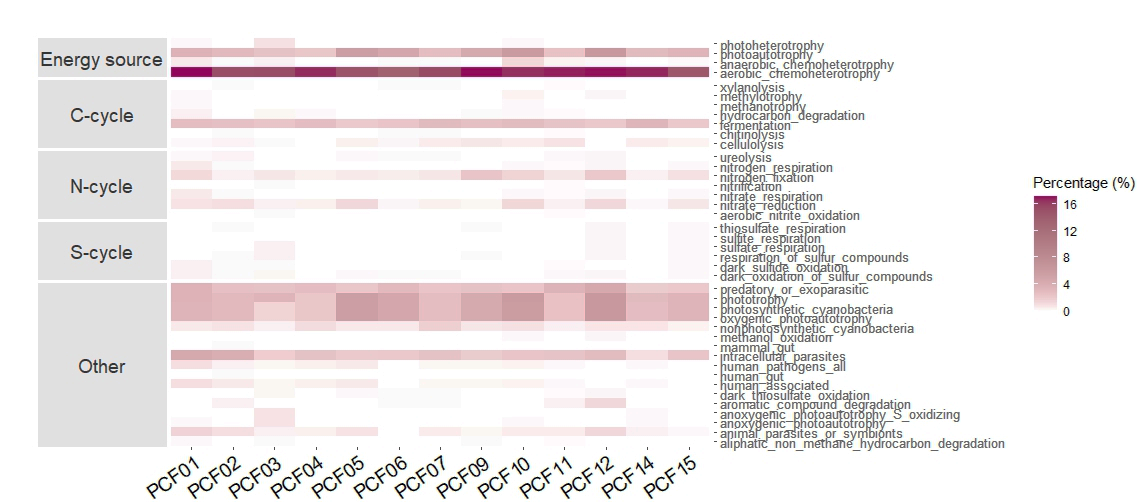
**

**Supplementary Fig. 3** Functional prediction of bacterial communities associated with *O. faveolata* lab-bred settlers, according to the FAPROTAX database.


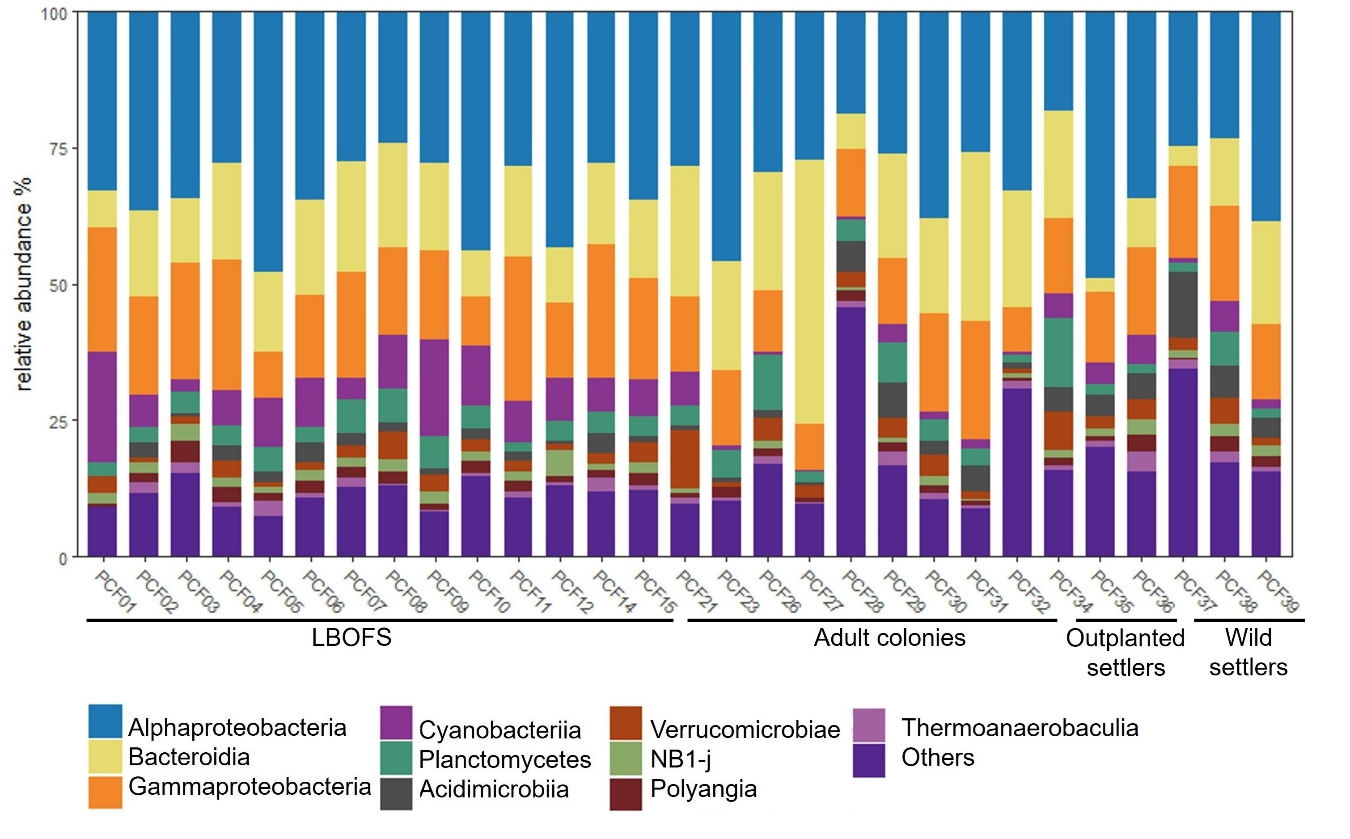


**Supplementary Fig. 4** Bacterial community composition at class level across all *O. faveolata* samples. Relative abundances of the 10 major bacterial classes are shown. Rare biosphere is grouped under “Others”.
